# Reform Should Make Health the First Item of Business

**DOI:** 10.1371/journal.pmed.0050208

**Published:** 2008-10-14

**Authors:** The PLoS Medicine Editors

## Abstract

The*PLoS Medicine* Editors argue that overcoming inequities and impediments to care in the US health care system will require directly addressing the prioritization of private profits over public health.

The past few months have seen a number of new studies finding the United States health care system—if indeed a patchwork of special interests, good intentions, and haphazard administrative requirements can honestly be called a system—to be growing ever more expensive, less efficient, and less accessible. The data also indicate that health care in the US has become increasingly inequitable.

An individual's access to medical care in the US is contingent on a multitude of factors. These include: (1) age, the major eligibility criterion for the national Medicare insurance program; (2) income and disability, factors considered in Medicaid programs supported by the federal government but administered differently by each state; (3) employment, through which some, but not all, workers obtain access to private insurance plans; and (4) specific diagnoses that may qualify patients for certain government health insurance plans while barring them from consideration by private plans. Another important factor is whether or not a patient's health plan approves a particular drug, medical procedure, or practitioner. Even assuming that one qualifies for and can afford a health insurance plan, the items covered by that plan are subject to change depending on business decisions by the plan's management, and plans often deny coverage of services that have already been provided, leaving the patient responsible for the full cost. The business logic of US health care also includes the stark fact that prohibitively high insurance premiums can effectively deny care to people with pre-existing illnesses; in other developed countries, such patients would have access to affordable health care guaranteed as a basic human right [1].

According to recent reports by the Commonwealth Fund [2,3], spending on health care in the US is double the amount per capita of other developed countries. And yet the US has little to show for the extra expense, having fallen to last place among 19 industrialized countries in terms of deaths that could have been prevented by timely and effective health care [2].

Indeed, despite this disproportionate spending, the Commonwealth Fund survey found that more than a third of US adults reported having gone without needed health care in 2007 because of costs. More than a quarter, or approximately 50 million people, were without health insurance for at least part of the previous year. Compared with 38 million people in 2001, this increase provides little endorsement of free market approaches to the problem [3].

Further, the survey found that 72 million (or 41% of) working-age adults faced serious financial problems from medical bills, which resulted in more than one-quarter becoming unable to pay for food, heat, or rent [3]. Ironically, these burdensome payments largely go to fund inefficiency, with health insurance administrative costs in the US running 30%–70% higher (as a proportion of total health spending) than in countries with more efficient public/private insurance systems (such as Germany, Switzerland, and the Netherlands). In other words, if the US could reduce these administrative costs to levels that European countries have already achieved, more than US$50 billion per year could be redirected to improve access to actual heath services [2].

Such improvements in access are desperately needed in the effort to address increasingly evident disparities in health and mortality within the US population. In purely financial terms, those with lower incomes pay disproportionately more for coverage, if they can pay at all. According to Commonwealth Fund data, half of families with incomes under US$20,000 were uninsured for at least part of 2007. Half of adults with annual income less than US$20,000 spent 10% or more of their income to cover health care costs or insurance premiums, compared with fewer than one in five adults with income more than US$60,000 [3].

The implications of high health care costs go beyond financial concerns. Under the current system, health insurance—affordable or not—remains a major determinant of access to care, notably among those with chronic diseases [4]. Alarmingly, recent studies in the US have found disparities in actual health outcomes between racial and ethnic groups, as well as socioeconomic groups, to be on the rise. One study published in *PLoS Medicine* found that gaps in mortality rates between rich and poor and between whites and populations of color narrowed between 1966 and 1980, but then remained stagnant (for infant mortality) or increased (for deaths before age 65) between 1981 and 2002 [5]. Another *PLoS Medicine* study similarly found that inequalities in life expectancy across US counties were at their lowest in 1983, after 20 years without significant mortality increases in any county, but that between 1983 and 1999, life expectancy declined significantly in 11 counties for men and in 180 counties for women. (Another 48 and 783 counties had statistically non-significant life expectancy declines in men and women, respectively.) Gains in life expectancy after 1983 were greater in counties with higher income, while declines in life expectancy were seen in counties with higher proportions of blacks [6]. The increasing disparities in mortality during the 1980s coincided with tax relief for the wealthy, cutting back of US public health and antipoverty programs, and worsening inequity in access to, and quality of, health care [5].

In election years, much is made of economic issues that affect quality of life. As we in open-access publishing have learned, it takes a good fight to change the status quo in a society that requires very strong evidence to place a public good above the individual interests of private enterprises that have established themselves as profitable. The same money identified as wasteful administrative costs also represents existing salaries and profits. Nonetheless, the evidence is now abundant and compelling that past failures to establish an equitable, accessible, and affordable health care system are coming home to afflict the lives of Americans by the tens of millions.

If Americans as a society truly value health and long life for the many, Americans must require limitations on market-driven health care to ensure that profit doesn't eclipse health in actual practice. Americans must also devote sufficient resources to public health care programs to enable these programs to compensate for the shortcomings of a for-profit system. In an essay written for a special collection of articles in *PLoS Medicine* devoted to social health (http://collections.plos.org/plosmedicine/socialmedicine-2006.php), the former United States Surgeon General David Satcher argued that to solve the problem of health disparities, “[W]e all must be proactive as advocates for change. The general public can work to improve access to quality care by advocating for universal access to such care. This means we must participate in the democratic process and elect representatives who will support legislation that ensures the availability of and access to quality care for all” [7].

Such elected officials will need to show initiative that goes beyond addressing the fragmented interests of individual businesses and organizations with stakes in the current, dysfunctional arrangement. John Iglehart, founding editor of the journal *Health Affairs* and national correspondent for the *New England Journal of Medicine*, recently commented regarding the role of physician organizations in Medicare planning: “because any viable plan is certain to result in both winners and losers, organized medicine, too, has been reluctant to act” [8]. America needs leaders, in government and elsewhere, who can see past short-term winning and losing to construct a system dedicated to meeting the health care needs of the people. The task is doubly complex in that even a system that offers universal access to care may still be characterized by health disparities among socioeconomic groups [9]. Nonetheless, accountability to an informed and increasingly affected public with free access to information on all aspects of the health care system should provide substantial motivation to competitive businesses and elected officials alike.

Solving the problems of access and inequity in American health care will require reworking the single-minded pursuit of individual financial gain that has been promoted in the US as an economic panacea since the early years of the Reagan administration, the same years in which the disparities noted above began to increase. American prosperity need not require a society in which the rich get richer as the poor get sick, and the sick become poor. As legal scholar Yochai Benkler has pointed out in the context of information exchange, collaboration “isn't the creation of some new utopian human type. We are as we have always been. Sometimes we do things for money. Sometimes we talk to friends or stop to give directions on the street. Sometimes we do things because they are the right things to do” [10].
